# Microencapsulation and Application of Probiotic Bacteria *Lactiplantibacillus plantarum* 299v Strain

**DOI:** 10.3390/microorganisms11040947

**Published:** 2023-04-05

**Authors:** Weizhe Sun, Quang D. Nguyen, Botond Kálmán Süli, Firas Alarawi, Anett Szécsi, Vijai Kumar Gupta, László Ferenc Friedrich, Attila Gere, Erika Bujna

**Affiliations:** 1 Department of Bioengineering and Alcoholic Drink Technology, Institute of Food Science and Technology, Hungarian University of Agriculture and Life Sciences, Ménesi út 45, H-1118 Budapest, Hungary; sun.weizhe@phd.uni-mate.hu (W.S.); nguyen.duc.quang@uni-mate.hu (Q.D.N.); sboti29@gmail.com (B.K.S.); firas.alarawi.94@gmail.com (F.A.); szecsi.anett@uni-mate.hu (A.S.); 2 Biorefining and Advanced Materials Research Center, Scotland’s Rural College (SRUC), Kings Buildings, West Mains Road, Edinburgh EH9 3JG, UK; vijaifzd@gmail.com; 3 Department of Livestock and Food Preservation Technology, Institute of Food Science and Technology, Hungarian University of Agriculture and Life Sciences, Ménesi út 45, H-1118 Budapest, Hungary; friedrich.laszlo.ferenc@uni-mate.hu; 4 Department of Post-Harvest Technology, Trade, Supply Chain and Sensory Evaluation, Institute of Food Science and Technology, Hungarian University of Agriculture and Life Sciences, Villányi út 29-43, H-1118 Budapest, Hungary

**Keywords:** microencapsulation, lyophilization, probiotics, *Lactiplantibacillus plantarum* 299v, polysaccharides, maltodextrin, resistant starch

## Abstract

Microencapsulation is an up-and-coming technology for maintaining the viability of probiotics. However, the effect of core-to-wall ratios and ratios of polysaccharides on the protection of the *Lactiplantibacillus plantarum* 299v strain has not been deeply discussed. Lyophilization of the *Lp. plantarum* 299v strain was conducted, and different core-to-wall ratios and ratios of maltodextrin (MD) and resistant starch (RS) were applied. Results demonstrated that the content of MD and RS had an influence on the yield and bulk density in both core-to-wall ratios (1:1 and 1:1.5). In addition, samples coated with a core-to-wall ratio of 1:1.5 had significantly higher viability than those coated with a core-to-wall ratio of 1:1. Moreover, samples coated with core-to-wall ratios of 1:1 and MD:RS 1:1, as well as core-to-wall ratios of 1:1.5 and MD:RS 3:1, had the highest cell number after simulated gastric fluid and simulated intestinal fluid testing, respectively. Furthermore, the optimal formulation for the application of microencapsulated *Lp. plantarum* 299v in apple juice (serving as a functional beverage) is listed as follows: core-to-wall ratios of 1:1 and MD:RS 1:1, with the fortification method, and stored at 4 °C. After 11 weeks of storage, the cell count was 8.28 log (CFU/mL). This study provided a strategy for *Lp. plantarum* 299v to achieve high viability in long-term storage and provides an application in functional apple beverages.

## 1. Introduction

With the improvement in living standards, a rising number of people have a higher prospect for foods in terms of nutritional balance and functionality [1,2]. For this reason, healthy, vegan, and fortified functional food, such as probiotic fortified fruit juice, has arisen and steadily taken substantial market share in recent years. Probiotics are delimited as “living microorganisms that can confer health benefits on the host when administrated in adequate number” [3]. The benefits of probiotics include nutritional functionality, regulating intestinal microbiota, weakening irritable bowel syndrome, inhibiting the growth of pathogenic bacteria, depressing blood pressure, lowering cholesterol levels, increasing immunity, and other physiological functions, e.g., reducing lactose intolerance illness, reducing the risk of colon cancer, improving constipation, regulating body weight, preventing tooth decay and delaying aging [4,5,6]. According to the World Health Organization (WHO) and the Food and Agriculture Organization (FAO), the recommended minimum number of product of probiotics is 10^6^ CFU/mL or CFU/g. Hence, the viability of probiotics and the dose level of probiotic products are the most significant factors when considering their function in the colon [7]. However, due to harsh factors, e.g., oxygen content, high acidity, and high temperature, during the production and storage process, and the low pH value and high bile salt concentration during the digestion process, it is difficult for probiotics to survive, let alone maintain the ideal viability and dose level after undergoing the digestive process [1,8,9,10,11]. 

Microencapsulation is a promising technology for maintaining the viability and functionality of probiotic strains during the process of manufacturing, storage, and digestion [5,6,12,13,14,15]. There are two aspects that need to be considered in the microencapsulation process. On the one hand, the type of microencapsulation method is a fundamental aspect, e.g., lyophilization [9,16,17], spray-drying [18,19], emulsification, and so on. Lyophilization is defined as a process that turns the moisture in the sample into ice and allows water to sublimate from a solid to a gaseous state under vacuum conditions, so as to achieve the purpose of dehydration, which is the most commonly used microencapsulation technology in the food and pharmaceutical industry. The advantages of lyophilization are that it is carried out at low temperatures, is processed under a vacuum condition, and has high water-moving ability, which can evade heat damage and avoid oxygen toxicity, thus resulting in longer shelf life [20]. On the other hand, the natural characteristics, functionality, and constructions of coating materials also need to be investigated. Polysaccharides such as maltodextrin [6,21] and resistant starch [6,22] are naturally produced and are GRAS (Generally Regarded as Safe) products, and they have been used as food additives in the food industry for a long time. Maltodextrin is a product produced by starch hydrolysis with high molecular weight, which has a good film-forming ability. Resistant starch is a small branch of starch, which has the properties to resist the hydrolysis by α-amylase and pullulanase in the saliva and stomach, but it can be fermented by probiotics in the colon [6]. Owing to the advantages of their physical properties, functional characteristics, and the fact that no toxic surfactant is added to the dispersion [23], maltodextrin and resistant starch were selected as the coating materials in this study. Research respecting the effects of singular polysaccharides as a coating material on the viability of probiotics is generally reported. However, the investigation of the comparison between two polysaccharides and their combination, especially the effect of core-to-wall ratios and ratios of wall materials, has not been deeply discussed.

The objective of this study was to develop and characterize the polysaccharide microcapsules as well as explore their application in the production of functional probiotic apple juice. 

## 2. Materials and Methods

### 2.1. Materials 

The *Lactiplantibacillus plantarum* 299v strain (Probi Corp., Lund, Sweden) was from the microorganism collection of the Department of Bioengineering and Alcoholic Drink Technology (Hungarian Agriculture and Life Sciences University). It was used as the core material in this study. High-purity maltodextrin (MD) and resistant starch (RS) were purchased from the Ingredion Company (Germany). High-quality unfiltered HAZÁNK Kincsei apple juice (Lidl, Hungary) was purchased from a local supermarket.

### 2.2. Microencapsulation of Probiotic Cells

The *Lactiplantibacillus plantarum* 299v strain was grown in de Man, Rogosa, and Sharpe (MRS) broth at 37 °C for 18 h and the cell count was around 10^9^ CFU/mL (Colony Forming Unit). Then, the cells were harvested by centrifugation at 10,000× *g* at 4 °C for 20 min and washed twice with phosphate-buffered saline (PBS) solution (0.1 M, pH 7.4). The formulation of the samples for encapsulation was made by mixing the wet pellet of the cells with wall materials, based on Table 1. Preparations were placed on a constant temperature shaker at 150 rpm at 25 °C for 1 h.

**Figure 1:**
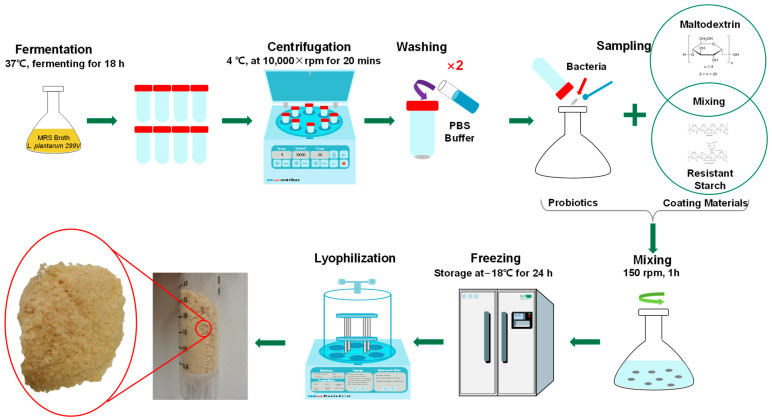
Scheme of microencapsulation process of *Lp. plantarum* 299v with carbohydrate.

The suspension of each sample was dispensed into a sterilized and clean drying bottle. Then, the samples were stored at −18 °C for 24 h before lyophilization (Figure 1). The appropriately prepared and well-mixed samples were lyophilized by a laboratory-scale lyophilization machine (Christ Alpha 2–4 Freeze Dryer, Martin Christ, Osterode am Harz, Germany). The process pressure and temperature were 0.250 mbar and 17 °C, respectively. The dried samples of microcapsules were ground manually under aseptic conditions, transferred into sterilized vials, and stored at 4 °C for future analysis.

### 2.3. Determination of Encapsulation Yield

The yield of the microencapsulation was evaluated by comparing the percentage of total solid materials before lyophilization to that after lyophilization. The weight of total solids before lyophilization includes wet bacteria and coating materials, while the weight of total solids after lyophilization contains only the dried probiotic microcapsules. The yield was calculated according to Equation (1).

(1) $$Y=\frac{m_{t}}{m_{0}}\times 100\%$$

where Y refers to the yield (%), m_0_ and m_t_ mean the total solid weight (g) before and after lyophilization, respectively [24,25].

### 2.4. Determination of Cell Number

The viable cell number was examined by the cell plate-counting method on MRS agar with 10-fold serial dilutions by using sterile 0.85% *w/w* sodium chloride solution. Generally, the colonies were determined after 48–72 h incubation [2,26]. The viable cell number of microcapsules was determined after completely releasing through disintegration in 0.85% saline solution. All enumerations were achieved in duplicate, and the plates containing 30–300 colonies were calculated and expressed as CFU/g of dried samples or CFU/mL of solution.

### 2.5. Determination of Bulk Density

Bulk density was analyzed by measuring the volume of 1g of sample in a 5 mL cylinder after being put on a vortex vibrator to tap for 2 min. The bulk density was defined according to Equation (2).

(2) $$\rho =\frac{m}{v}\times 100\%$$

where ρ is the bulk density (kg/m^3^), m is the weight (kg) of the sample, and v is the volume (m^3^) of the sample in the cylinder [25,26].

### 2.6. Scanning Electron Microscopy 

The morphologies of microcapsules were examined by scanning electron microscopy (SEM, Thermo ScientificTM PrismaTM E, Waltham, MA, USA) under high vacuum conditions, with an augmented voltage of 15 kV. The samples were stuck on a plate in the vacuum chamber and gradually decreased to 200 Pa before the examination. The microcapsules were viewed at 1000× and 14,000× magnifications [26].

### 2.7. Viability of Microencapsulated Lp. plantarum 299v during Storage

The viability of microencapsulated probiotics during storage at 4 °C and 25 °C was investigated. An appropriate number of microcapsules was put into bottles and then all bottles were placed either in the refrigerator (4 °C) or incubator (25 °C). Samples were taken every week for 8 weeks, and the viable cells were determined by plate-counting method. Different regressed models were used to fit the changes in cell numbers during the storage time.

### 2.8. Tolerance of Microencapsulated Probiotics to SGF and SIF

The tolerance of microencapsulated probiotics to simulated gastric fluid (SGF) and simulated intestinal fluid (SIF) was checked in vitro according to the method described previously [26,27,28]. 

### 2.9. Application of Probiotic Microcapsules in Apple Juice 

The application potential of probiotic microcapsules encapsulated with polysaccharides was checked using high-quality unfiltered HAZÁNK Kincsei apple juice. Both fermentation and fortification technologies were applied. The pH of apple juice was adjusted to pH 6 by 4 N NaOH solution for future utilization. Three types of probiotic microcapsules were coated and used: with MD in the core-to-wall ratio of 1:1, with MD:RS 1:1 in the core-to-wall ratio of 1:1, and with RS in the core-to-wall ratio of 1:1 (namely SA1, SA2, and SA3, respectively). Ninety milliliters of apple juice were transferred into 250 mL bottles and then 0.2 g microcapsules (the cell number were 10.73, 10.82, 10.12 log (CFU/g) for SA1, SA2, and SA3, respectively) were added and suspended well. In the case of fermentation, the experimental runs were incubated at 37 °C for one day. In the case of fortification, no fermentation process was carried out. Then, in both cases, the apple juices were divided into two parts. One was stored at 4 °C and the other at 25 °C for 8 weeks. The samplings were conducted in time intervals of one week.

### 2.10. Statistical Analysis

All experiments were performed in duplicate, and the results are presented as means ± standard deviation. ANOVA (analysis of variance) with a significance level of α = 0.05 was used to determine statistical differences among the independent variables by using SPSS AU (www.spssau.com/en, accessed on 19 October 2022).

## 3. Results and Discussions

### 3.1. Yield of Microencapsulation Process

**Figure 2:**
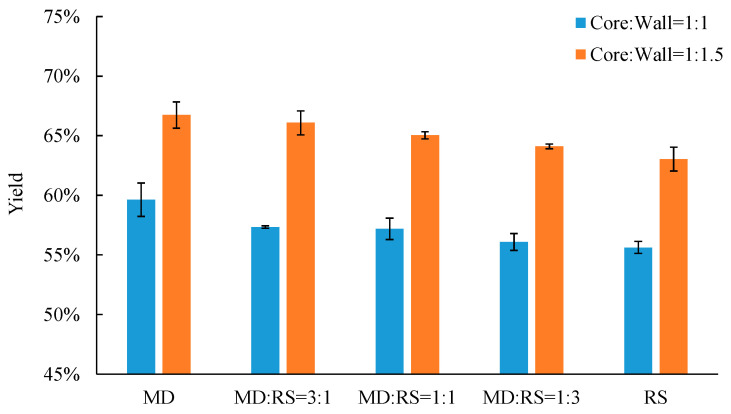
Yield of microencapsulated *Lp. plantarum* 299v with different core-to-wall ratios and ratios of wall materials. MD— maltodextrin, RS— resistant starch.

Yield is the fundamental parameter that is needed during the production, packaging, and storage process. The yields of the microencapsulation process of the probiotic *Lp. plantarum* 299v strain with different core-to-wall ratios and ratios of wall materials are shown in Figure 2. The yield varied from 55.63% to 59.63% with a core-to-wall ratio of 1:1 and from 63.04% to 66.74% with a core-to-wall ratio of 1:1.5. The decrease in maltodextrin content (or increase in resistant starch content) resulted in a decrease in yield in both core-to-wall ratios, of 1:1 and 1:1.5. The results displayed that the ratios of wall materials had a significant influence on the yield. The reason may be due to the natural characteristics of maltodextrin, resulting in spongy microcapsules that have more water molecules remaining [14,29]. In addition, samples coated with core-to-wall ratios of 1:1.5 have a significantly higher yield than those coated with core-to-wall ratios of 1:1. The reason may be due to the higher weight of coating materials of samples with core-to-wall ratios of 1:1.5 than those coated with core-to-wall ratios of 1:1.

### 3.2. Cell Number and Bulk Density of Microcapsules

**Figure 3:**
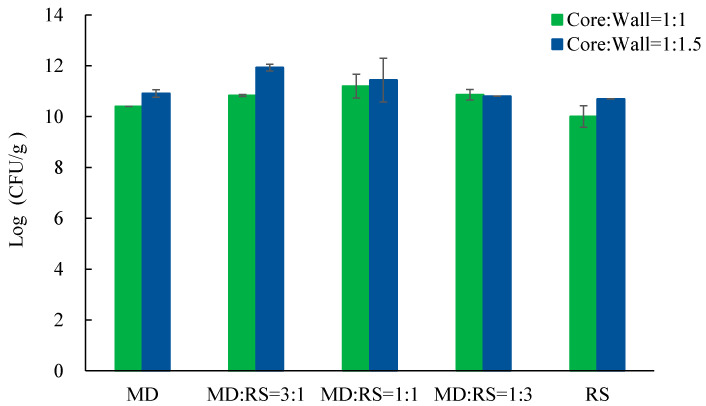
Cell number of microencapsulated *Lp. plantarum* 299v with different core-to-wall ratios and ratios of wall materials. MD— maltodextrin, RS— resistant starch.

Viable cell number, or viability, is the most important indicator of probiotics in probiotic products, which can indicate the quality of probiotic products. The cell numbers of the microcapsules of probiotic *Lp. plantarum* 299v with different core-to-wall ratios and ratios of wall materials are shown in Figure 3.

Based on the requirements and suggestions of the FAO/WHO, the minimal cell number of probiotic products should be higher than 6 log (CFU/g) or 6 log (CFU/mL). Some researchers suggest that this number should be increased to 7 log (CFU/g) [30]. Nevertheless, most researchers have already used the microencapsulation method to achieve the minimum requirements with 6 log (CFU/g) or 7 log (CFU/g). Different types of porous maize starches [9] and skim milk [31] were applied as coating materials to microencapsulate *Lp. plantarum* 299v and *Lactobacillus gasseri* CRL1421 by the freeze-drying method, and microcapsules were achieved with a cell count of 9.21 log (CFU/g) and 10.89 log (CFU/g), respectively. In our case, the cell number varied from 10.01 log (CFU/g) to 11.93 log (CFU/g) (Figure 3). The highest cell number of our microcapsules was considerably larger than the ones reported [9,29]. In particular, samples coated with core-to-wall ratios of 1:1 and MD:RS 1:1, core-to-wall ratios of 1:1.5 and MD:RS 3:1, and core-to-wall ratios of 1:1.5 and MD:RS 1:1 all had a cell number greater than 11 log (CFU/g), which were 11.19 log (CFU/g), 11.93 log (CFU/g), and 11.43 log (CFU/g), respectively. Therefore, in the real industrial production of probiotic products, more excipient materials can be added, or fewer microcapsules are needed, in order to save on production costs while still achieving a relatively high probiotic cell number. Moreover, probiotics coated with the core-to-wall ratio of 1:1.5 have significantly higher viability than probiotics coated with the core-to-wall ratio of 1:1. One possible implication of this is that, relatively, more coating materials have a better-protecting ability during lyophilization, e.g., the dual replacement of sites for water molecules and dual formation of protective coating for probiotics during the drying process, respectively. Similar results were obtained by Rajam and co-workers (2015), who microencapsulated the *Lactobacillus plantarum* MTCC 5422 strain with fructooligosaccharide by spray-drying.

**Figure 4:**
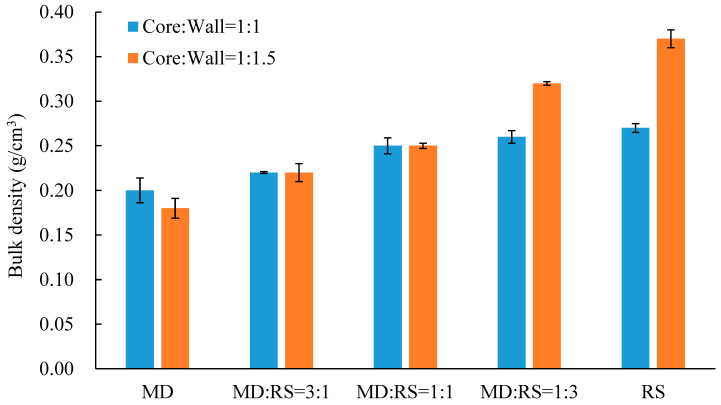
Bulk density of microencapsulated *Lp. plantarum* 299v with different core-to-wall ratios and ratios of wall materials. MD—maltodextrin, RS—resistant starch.

Bulk density is a physical property of the powder of microcapsules, which can affect the storage ability and solubility of the microcapsules [29,32]. The bulk densities of microcapsules of the probiotic *Lp. plantarum* 299v strain, with different core-to-wall ratios and ratios of wall materials, are shown in Figure 4.

The bulk density ranged from 0.20 kg/m^3^ to 0.27 kg/m^3^ with the core-to-wall ratio of 1:1 and from 0.18 kg/m^3^ to 0.37 kg/m^3^ with the core-to-wall ratio of 1:1.5, respectively. The results demonstrated that the core-to-wall ratios and ratios of wall materials have a significant influence on bulk density. The decrease in maltodextrin content (or increase in resistant starch content) resulted in an increase in the bulk density for both core-to-wall ratios, of 1:1 and 1:1.5. The reason may be due to the natural characteristics of maltodextrin and resistant starch, resulting in spongy microcapsules [14,29]. Fuchs and co-workers (2006) reported the close relationship between bulk density and microcapsules, i.e., a higher bulk density value indicates a higher weight in unit volume, which means small particle size. This characteristic also has a connection with the easy solubility of the powders. Hence, the bulk density of the microcapsules has some reference value for their application in the functional beverage.

### 3.3. Morphology of Microencapsulated Lp. plantarum 299v Strain

**Figure 5:**
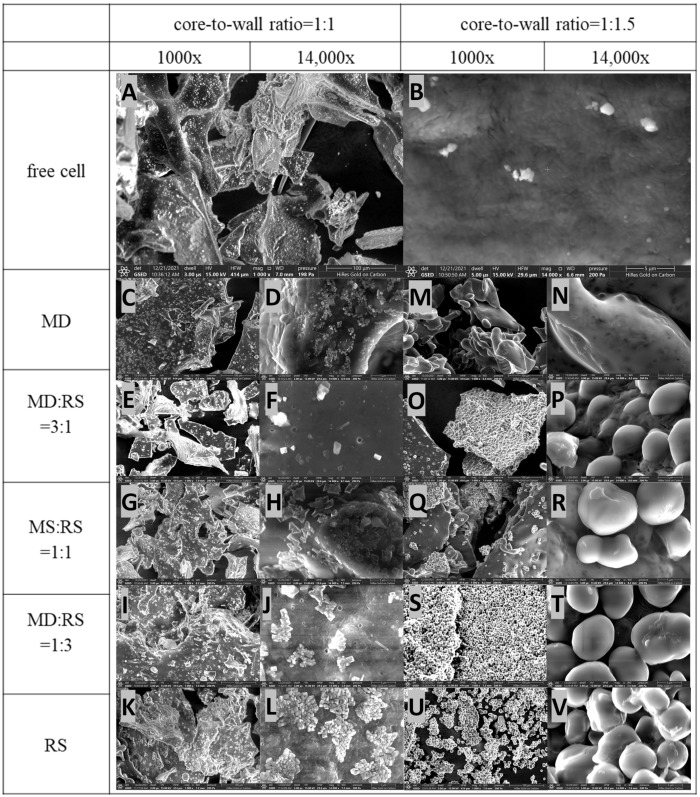
Scanning electron microscope (SEM) images of *Lp. plantarum* 299v cells under 1000× and 14,000× magnification. (**A**,**B**): free cells; (**C**,**D**): core-to-wall ratios of 1:1 and MD; (**E**,**F**): core-to-wall ratios of 1:1 and MD:RS 3:1; (**G**,**H**): core-to-wall ratios of 1:1 and MD:RS 1:1; (**I**,**J**): core-to-wall ratios of 1:1 and MD:RS 1:3; (**K**,**L**): core-to-wall ratios of 1:1 and RS; (**M**,**N**): core-to-wall ratios of 1:1.5 and MD; (**O**,**P**): core-to-wall ratios of 1:1.5 and MD:RS 3:1; (**Q**,**R**): core-to-wall ratios of 1:1.5 and MD:RS 1:1; (**S**,**T**): core-to-wall ratios of 1:1.5 and MD:RS 1:3; (**U**,**V**): core-to-wall ratios of 1:1.5 and RS. MD: maltodextrin; RS: resistant starch.

Scanning electron microscope (SEM) images can provide detailed information about the surface topography of the sample. Microscopy pictures of free cells of *Lp. plantarum* 299v are demonstrated in Figure 5A,B. Images of the microstructure of microencapsulated *Lp. plantarum* 299v with different core-to-wall ratios and wall material formulations are shown in Figure 5C–V.

Figure 5A,B shows pictures of free *Lp. plantarum* 299v cells under 1000× and 14,000× magnification, respectively. It is obvious that the cells are clustered together, and the rod-shaped cells can be seen under 14,000× magnification. In addition, all the 14,000× magnification images demonstrated that the rod-shaped *Lp. plantarum* 299v cells were homogenously microencapsulated and covered by the coating materials. Moreover, the results of microencapsulated samples (Figure 3C–V) are in accordance with the conventional observation of matrix-type microcapsules—that the cells are dispersed in the coating materials while they may present on the surface of the materials [8,33]. This means that the probiotics are randomly distributed over the surface and within the microcapsules. In addition, the surface of microcapsules is smoother, and the structure is more uniform in samples with a core-to-wall ratio of 1:1.5 than in microcapsule samples with a core-to-wall ratio of 1:1. Furthermore, several tiny cracks and holes on the microcapsules were visible at higher magnifications in samples with a core-to-wall ratio of 1:1, while there was no similar phenomenon in the samples with a core-to-wall ratio of 1:1.5 or samples with free cells. The reason for these properties may be due to the increasing content of coating materials, especially the resistant starch content, which exposed more starch granules on the surface [8]. Meanwhile, it also resulted in a better protecting ability due to the thicker coating materials, which not only work as protective barriers against the harsh environment of probiotic cells but also have the function of preventing water uptake [34].

### 3.4. Viability of Microencapsulated Lp. plantarum 299v during Storage

**Figure 6:**
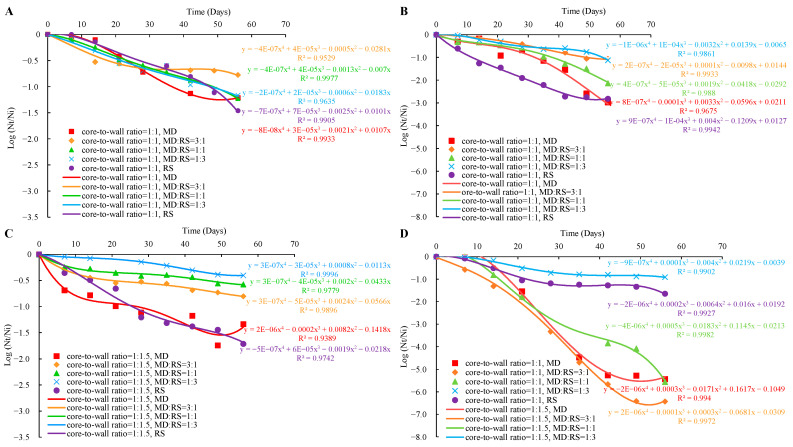
Viability loss of microencapsulated *Lp. plantarum* 299v with different core-to-wall ratios and ratios of wall materials at 4 °C and 25 °C. (**A**): 4 °C, core-to-wall ratio of 1:1; (**B**): 25 °C, core-to-wall ratio of 1:1; (**C**): 4 °C, core-to-wall ratio of 1:1.5; (**D**): 25 °C, core-to-wall ratio of 1:1.5; MD: maltodextrin; RS: resistant starch.

The effectiveness of different core-to-wall ratios and ratios of wall materials on the storage stability of the microencapsulated *Lp. plantarum* 299v strain, stored at 4 °C and 25 °C for 8 weeks, is demonstrated in Figure 6.

In the case of samples stored at 4 °C with the core-to-wall ratio of 1:1 (Figure 6A), probiotics coated with MD:RS 1:1, MD:RS 1:3, and RS had almost the same constant viability loss rate. The only difference was that in the case of probiotics coated with RS the viability decreased sharply from the end of the sixth week. The viability loss of probiotics coated with MD started from the first week to the sixth week and then remained constant. Moreover, it was a similar trend for probiotics coated with MD:RS 3:1. However, the unchanged state started from the fourth week. These results might be due to the probiotics coated with MD and MD:RS 3:1 being well protected by the coating materials with a dense structure. Only the probiotics exposed outside of capsules were oxidized during the first 6 weeks and 4 weeks of storage. In the case of samples stored at 4 °C with the core-to-wall ratio of 1:1.5 (Figure 6C), the viability loss of probiotics coated with a mixture of MD and RS was significantly lower than probiotics coated with a single material.

In the case of storage at 25 °C with a core-to-wall ratio of 1:1 (Figure 6B), probiotics coated with MD:RS 3:1 had a constant viability loss rate for 8 weeks of the experiment. Similarly, probiotics coated with MD:RS 1:3, MD:RS 1:1, and MD had a constant viability loss rate in the previous weeks. However, from the fourth week and the seventh week, the viability of probiotics coated with MD:RS 1:1, MD, and MD:RS 1:3 decreased sharply, respectively. The viability loss rate of probiotics coated with RS was almost constant until the seventh week. This remained unchanged from the seventh to the eighth week. The same explanations can be addressed here that the formation of a dense structure after several weeks of oxidation and absorption of water molecules that changed the structure of the microcapsules. In the case of storage at 25 °C with the core-to-wall ratio of 1:1.5 (Figure 6D), probiotics coated with MD, MD:RS 3:1, and MD:RS 1:1 decreased the viability sharply in the first 7 weeks. From the seventh to the eighth week, the viability of probiotics coated with MD:RS 1:1 decreased even more seriously than in the previous 7 weeks, while the viability of probiotics coated with MD and MD:RS 3:1 remained unchanged. Moreover, the viability loss rate of probiotics coated with MD:RS 1:3 and RS almost remained constant and was significantly lower than that of the three samples of MD, MD:RS 3:1, and MD:RS 1:1. These results may be due to the RS content having a positive effect on the protecting ability of the probiotics under the circumstance of a core-to-wall ratio of 1:1.5 during long-term storage at 25 °C. 

In summary, all microencapsulated probiotics lost viability during the storage. However, the viability changing pattern of *Lp. plantarum* 299v is different depending on the coating material and core-to-wall ratios, as well as the ratios of wall materials. This indicates that the compactness and oxidation resistance of each microcapsule are quite different, even during the different storage stages, because some microcapsules can form dense structures even after several weeks of storage. Lower storage temperature can decrease the oxidation rate of the cell membrane, thus decreasing the viability loss rate.

### 3.5. Tolerance of Microencapsulated Probiotics to SGF and SIF

**Figure 7:**
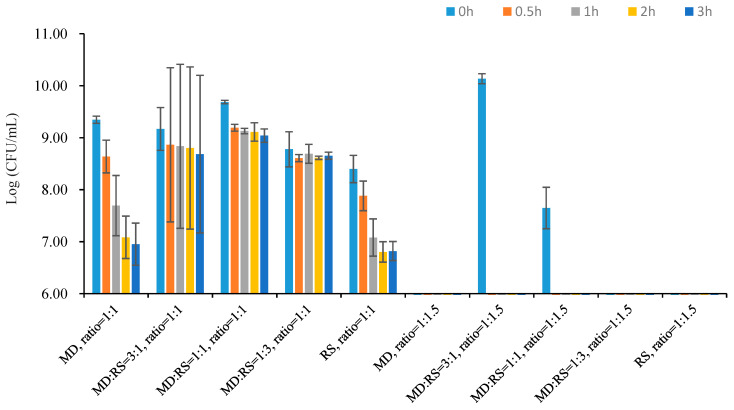
Viability of microencapsulated *Lp. plantarum* 299v with different core-to-wall ratios and ratios of wall materials after being exposed to SGF at 37 °C for 3 h. MD: maltodextrin; RS: resistant starch.

**Figure 8:**
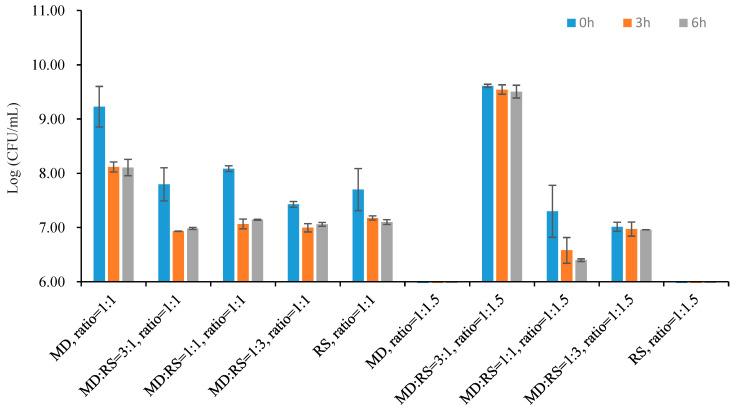
Viability of microencapsulated *Lp. plantarum* 299v with different core-to-wall ratios and ratios of wall materials after being exposed to SIF at 37 °C for 6 h. MD: maltodextrin; RS: resistant starch.

Harsh factors such as low pH value and high bile salt content are harmful to probiotics. Therefore, the ability of the microencapsulated probiotics to be resistant to the severe environment is a crucial property of the microcapsules [28]. The tolerance of microencapsulated *Lp. plantarum* 299v with different core-to-wall ratios and ratios of wall materials to SGF and SIF are shown in Figure 7 and Figure 8.

The stomach is the main organ and place of digestion of human beings. The acidity of the stomach is around pH 2, which is extremely harmful to probiotics. The viability of microencapsulated *Lp. plantarum* 299v with different core-to-wall ratios and ratios of wall materials, after being exposed to SGF at 37 °C for 3 h, is shown in Figure 7. The results demonstrated that most of the samples showed a loss of viability during the digestion process. This is caused by low pH and high acidity [25,28,35]. The core-to-wall ratio of 1:1 may have a better protectability compared to the core-to-wall ratio of 1:1.5. This is because most of the samples with the core-to-wall ratio of 1:1.5 cannot resist the SGF test. Only samples coated with MD:RS 3:1 and MD:RS 1:1 can last for a short time at the beginning of the simulated digestion process. In addition, the average reduction levels of samples with the core-to-wall ratio of 1:1 after 3 h of SGF digestion were 2.39 log (CFU/g), 0.48 log (CFU/g), 0.65 log (CFU/g), 0.12 log (CFU/g), and 1.58 log (CFU/g), respectively. The final cell numbers after 3 h of SGF digestion were 6.95 log (CFU/g), 8.68 log (CFU/g), 9.04 log (CFU/g), 8.65 log (CFU/g), and 6.82 log (CFU/g), respectively. Furthermore, samples coated with MD:RS 3:1, MD:RS 1:1 and MD:RS 1:3 had higher survival ability than samples coated with only MD or RS. This leads us to conclude that the combination of the coating materials resulted in better resistance to simulated gastric fluid than single-coating materials.

The tolerance of the probiotic microcapsules to the bile salt environment is a significant property [28,35]. Therefore, the viability of microencapsulated *Lp. plantarum* 299v with different core-to-wall ratios and ratios of wall materials, after being exposed to SIF at 37 °C for 6 h, is summarized in Figure 8. The results illustrated that the samples with the core-to-wall ratio of 1:1 can better tolerate bile salt than samples with the core-to-wall ratio of 1:1.5. Additionally, the average reductions in the viable cells of samples coated with the core-to-wall ratio of 1:1 were 1.12 log (CFU/g), 0.81 log (CFU/g), 0.94 log (CFU/g), 0.37 log (CFU/g), and 0.60 log (CFU/g), respectively. However, the sample coated with the core-to-wall ratios of 1:1.5 and MD:RS 3:1 had the highest cell number of 9.51 log (CFU/g), with only a 0.11 log (CFU/g) reduction after 6 h of SIF digestion. The difference may be due to the core-to-wall ratios and ratios of wall materials [17,18,22,25]. The final cell numbers after 6 h of SIF testing were 8.11 log (CFU/g), 6.98 log (CFU/g), 7.14 log (CFU/g), 7.06 log (CFU/g), and 7.10 log (CFU/g), respectively.

### 3.6. Application of Probiotic Microcapsules in Apple Juice 

**Figure 9:**
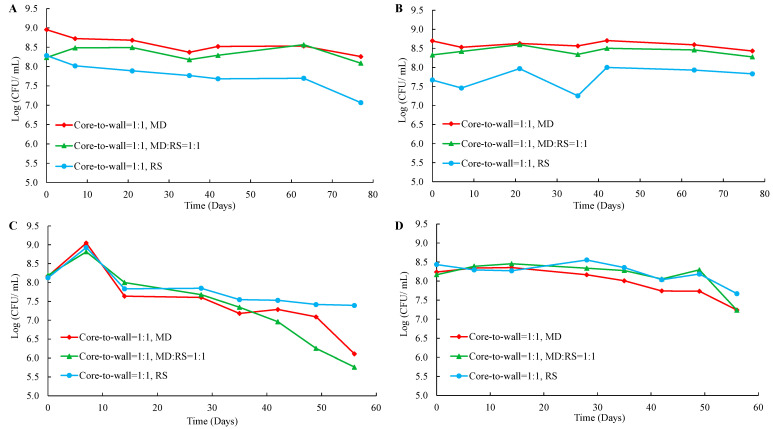
Viability of microencapsulated *Lp. plantarum* 299v in fermented and fortified apple juice at 4 °C for 11 weeks and 25 °C for 8 weeks. MD: maltodextrin, RS: resistant starch; (**A**): 4 °C, Fermentation; (**B**): 4 °C, Fortification; (**C**): 25 °C, Fermentation; (**D**): 25 °C, Fortification.

The stability of microencapsulated cells is quite different from diverse food matrices; even for the same kind of food matrices, the properties will change after the fermentation process. Hence, the stability of the microencapsulated cells in food matrices not only related to the characteristics of the food matrices but also to the coating materials [36]. In our research, the viability of microencapsulated *Lp. plantarum* 299v with different core-to-wall ratios and ratios of wall materials, in fermented and fortified apple juice at 4 °C for 11 weeks and 25 °C for 8 weeks, was investigated (Figure 9).

After 8 weeks of storage, the viabilities of the samples coated with core-to-wall ratios of 1:1 and MD:RS 1:1 that were stored at 25 °C and underwent a fermentation process were lower than 6 log (CFU/g). Moreover, the samples stored at 4 °C for 11 weeks had significantly higher viability than the samples stored at 25 °C for 8 weeks. The results suggest that temperature greatly affects maintaining the viability of the microencapsulated probiotics [20]. There is significantly higher viability in the fortified probiotic apple juice than the fermented probiotics stored at the same temperature. This means that the fortification method is suitable for maintaining the viability of probiotics in the apple juice. Furthermore, the viability of the probiotics in the fortified juices that were stored at 4 °C and coated with core-to-wall ratios of 1:1 and MD and the core-to-wall ratio of MD:RS 1:1 was higher than of those coated with RS. The reduction in the viability of the sample coated with core-to-wall ratios of 1:1 and MD:RS 1:1 was lower than that of the probiotics coated with core-to-wall ratios of 1:1 and MD. Zhang and co-workers (2020) suggested the use of 5.0% (*w/v*) lactose, mannitol, trehalose, ascorbic acid, gelatin and 10.0% (*w/v*) skim milk as a mixture to provide the optimal protecting composition by freeze-drying for *Pseudoalteromonas nigrifaciens*. In summary, the suitable conditions for the application of *Lp. plantarum* 299v in apple juice, in order to achieve the goal of high survival ability in this functional beverage, are core-to-wall ratios of 1:1 and MD:RS 1:1 for the coating material, a fortification process, and storage at 4 °C. After 11 weeks of storage, the cell count was 8.28 log (CFU/mL).

## 4. Conclusions

The polysaccharides maltodextrin and resistant starch are good coating materials for the microencapsulation of the *Lp. plantarum* 299v strain, and microcapsules were developed successfully by lyophilization. The yield of the encapsulation process as well as the cell number and bulk density of microcapsules were influenced by the core-to-wall ratios and the ratios of wall materials used. In addition, the viabilities of microencapsulated *Lp. plantarum* 299v during storage were affected by the core-to-wall ratios, the ratios of wall materials, the storage temperature, and time. Moreover, these factors also affect the tolerance of microencapsulated *Lp. plantarum* 299v to SGF and SIF. Furthermore, the changes in the viability of probiotic apple juices were influenced by core-to-wall ratios, ratios of wall materials, storage time, and temperature, as well as the fermentation or fortification technology applied. Overall, this study provides valuable insights into the development of effective probiotic delivery systems through microencapsulation with polysaccharides, and the newly developed microcapsules have high application potential in the fortification of apple juice. 

## Figures and Tables

**Table 1:** Experimental plan for microencapsulation of *Lp. plantarum* cells.

Core-to-Wall Ratios	Wall Materials Formulation	Ratios of Wall Materials (*w/w*)	MD (g)	RS (g)	*Lp. plantarum* 299v (Wet Weight, g)	Sample Solution Concentration (% *w/w*)
1:1	MD	-	10.00	0.00	10.00	20.00
	MD + RS	3:1	7.50	2.50	10.00	20.00
	MD + RS	1:1	5.00	5.00	10.00	20.00
	MD + RS	1:3	2.50	7.50	10.00	20.00
	RS	-	0.00	10.00	10.00	20.00
1:1.5	MD	-	15.00	0.00	10.00	20.00
	MD + RS	3:1	11.25	3.75	10.00	20.00
	MD + RS	1:1	7.50	7.50	10.00	20.00
	MD + RS	1:3	3.75	11.25	10.00	20.00
	RS	-	0.00	15.00	10.00	20.00

MD—Maltodextrin; RS—Resistant Starch.

## Data Availability

Not applicable.
